# Single-cell RNA-seq reveals T cell exhaustion and immune response landscape in osteosarcoma

**DOI:** 10.3389/fimmu.2024.1362970

**Published:** 2024-04-02

**Authors:** Qizhi Fan, Yiyan Wang, Jun Cheng, Boyu Pan, Xiaofang Zang, Renfeng Liu, Youwen Deng

**Affiliations:** ^1^Department of Spine Surgery, Third Xiangya Hospital, Central South University, Changsha, China; ^2^Department of Orthopedics, Third Hospital of Changsha, Changsha, China

**Keywords:** osteosarcoma, T cell exhaustion, tumor immune microenvironment, prognosis, immunotherapy

## Abstract

**Background:**

T cell exhaustion in the tumor microenvironment has been demonstrated as a substantial contributor to tumor immunosuppression and progression. However, the correlation between T cell exhaustion and osteosarcoma (OS) remains unclear.

**Methods:**

In our present study, single-cell RNA-seq data for OS from the GEO database was analysed to identify CD8+ T cells and discern CD8+ T cell subsets objectively. Subgroup differentiation trajectory was then used to pinpoint genes altered in response to T cell exhaustion. Subsequently, six machine learning algorithms were applied to develop a prognostic model linked with T cell exhaustion. This model was subsequently validated in the TARGETs and Meta cohorts. Finally, we examined disparities in immune cell infiltration, immune checkpoints, immune-related pathways, and the efficacy of immunotherapy between high and low TEX score groups.

**Results:**

The findings unveiled differential exhaustion in CD8+ T cells within the OS microenvironment. Three genes related to T cell exhaustion (RAD23A, SAC3D1, PSIP1) were identified and employed to formulate a T cell exhaustion model. This model exhibited robust predictive capabilities for OS prognosis, with patients in the low TEX score group demonstrating a more favorable prognosis, increased immune cell infiltration, and heightened responsiveness to treatment compared to those in the high TEX score group.

**Conclusion:**

In summary, our research elucidates the role of T cell exhaustion in the immunotherapy and progression of OS, the prognostic model constructed based on T cell exhaustion-related genes holds promise as a potential method for prognostication in the management and treatment of OS patients.

## 1 Introduction

Osteosarcoma (OS) is the most prevalent aggressive bone tumor occurring in children and adolescents, constituting the majority of all bone tumor cases (1). The conventional approach to OS entails a blend of surgery and rigorous multi-agent chemotherapy. However, the prognosis for OS patients remains exceedingly grim, primarily attributed to delayed diagnosis and early-stage distant metastasis (2). Therefore, it is crucial to explore innovative and efficacious therapies aimed at enhancing the prognosis of OS patients.

Immunotherapy holds significant promise in the treatment of malignant tumors in humans, numerous recent studies have highlighted its considerable potential in tumor therapy, with preclinical trials providing robust support (3). Immune checkpoint inhibitors (ICIs) exhibit considerable potential for immunotherapy in OS. They can navigate the genomic complexity of OS, leading to enhanced overall outcomes (4). Although immunotherapy for OS has demonstrated promising therapeutic effects in some studies, it has yet to substantially improve patient prognosis (5, 6). In clinical trials of OS, the response to ICIs has not been favorable and trial results are not yet satisfactory (7). This may be attributed to the immune microenvironment in OS, which suppresses T cell function (8). Immune cells constitute the cellular foundation of immunotherapy, of which CD8^+^ T cells serving as a pivotal component of cancer immunotherapy (9). Activated CD8^+^ T cells mature into cytotoxic T lymphocytes (CTLs) and represent a key component of the immune system’s antitumor response, CTLs are associated with increased survival rates in various types of cancer and play a crucial role in immune surveillance, targeting and eliminating cancer cells (10). The optimal approach for achieving tumor eradication will likely entail a combination of therapies that promote immune activation and T cell initiation, counteract immunosuppressive signals in the tumor microenvironment, and sustain the presence of T cells in cancerous tissue.

T cell exhaustion entails a progressive, hierarchical, and negatively regulatory process affecting T cells within the tumor microenvironment (11). Classical inhibitors targeting PD-1 and CTLA-4 largely exert their anti-tumor effects by mitigating functional exhaustion (12). However, the precise underlying mechanisms of these inhibitors necessitate further investigation. The recent advancement of biomarkers unveiled potential molecular regulatory targets for CD8^+^ T cells in the intricate tumor heterogeneity of OS. Moreover, the potential correlation between alterations in exhaustion expression profiles and immune checkpoints has presented avenues for research (13).

In this study, we aim to delve into potential molecular regulatory targets and core regulatory genes associated with T cell exhaustion in the intricate tumor heterogeneity of OS. We developed a multi-biomarker model based on genes linked to T cell exhaustion, which functions in evaluating the tumor microenvironment, predicting immunotherapy response, and forecasting the prognosis of diverse OS patients. It has great potential to play a vital role in guiding clinical practice in the future.

## 2 Methods

### 2.1 Obtaining the raw data

The single-cell sequencing data (GSE162454), along with microarray data (GSE16091 and GSE21257) pertaining to OS, were acquired from the GEO database (http://www.ncbi.nlm.nih.gov/geo). Additionally, data from 84 distinct OS patients’ samples were retrieved from the TARGETs database. All datasets were accompanied by clinical and prognostic information, which was employed for subsequent analyses.

### 2.2 Data processing of single-cell RNA sequence

Data analysis and quality assessment were conducted using the R package “Seurat” (version 4.3.0; http://satijalab.org/seurat/). Cells with expression of fewer than 250 genes or with a percentage of mitochondrial genes exceeding 20% of the total expressed genes were excluded from the analysis. Additionally, cells with unique molecular identifiers (UMI) resulting in log10(UMI) > 0.80 were also removed. Subsequently, potential doublets were identified and eliminated using the R package “DoubletDecon” (version 1.1.6; http://EDePasquale/DoubletDecon).

### 2.3 Data integration and dimensionality reduction

The feature counts for each cell underwent a transformation, involving division by the total counts for that cell, followed by multiplication by 10,000. Subsequently, the results were logarithmically transformed and then normalized by adding 1, thus preventing the computation of the logarithm of 0. Before proceeding with the normalization of the expression matrix, the top 2000 highly variable genes (HVGs) were identified, centered, and scaled. Subsequent to this, a principal component analysis (PCA) was conducted based on these HVGs. Following that, the R package “Harmony” (https://github.com/immunogenomics/Harmony) was employed to integrate the cellular data from six samples and mitigate any potential batch effects.

### 2.4 Cell-clustering and annotation

The clustering analysis relied on the embedding of the Harmony algorithm, executed through the “FindNeighbors” and “FindClusters” functions within the “Seurat” package. The resulting clusters were visualized on a two-dimensional plot generated via the UMAP method. For subcluster analysis, akin procedures were applied, encompassing variable gene identification, dimensionality reduction, Harmony for cell integration, and cluster identification for the distinct clusters derived from the overall analysis. The annotation of clusters was performed using established cellular markers drawn from the literature. Detailed information regarding the cellular markers can be found in the **Supplementary Tables**.

### 2.5 Identification and analysis of CD8^+^ T cell subtypes

CD8^+^ T cells were isolated and subsequently re-clustered using the “Seurat” package in R. Single-cell pseudotime trajectories were constructed employing the “Monocle2” package in R. Following this, a weighted correlation network analysis (WGCNA) was conducted to identify the core gene sets within CD8^+^ T cellular clusters using the “hdWGCNA” package in R. To explore intercellular communication between all cell clusters, the R package “Cellchat” was utilized. The levels of immune checkpoint molecules between clusters were assessed based on the immune checkpoint expression profile. Differential functional status regarding Gene Ontology (GO) and KEGG pathways for each CD8^+^ T cell cluster were analyzed using the “ClusterProfiler” R package. Additionally, the GSEA pathways obtained from MSigDB (gsea-msigdb.org) were evaluated using the “fgsea” R package. Furthermore, differences in HALLMARK pathways between the clusters were determined through gene set variation analysis (GSVA) using the “GSVA” R package.

### 2.6 Construction and validation of the T cell exhaustion signature

The CD8^+^ T cell exhaustion genes with prognostic potential in the TARGETs dataset were identified through Univariate Cox regression analysis (P<0.05). Subsequently, a combination of six machine learning algorithms was employed, which included the least absolute shrinkage and selection operator (LASSO) Cox regression algorithm (14), Boruta feature selection algorithm (15), survival support vector machine (survival-SVM) based on 10-fold cross-validation (16), Boosting in Cox regression (Cox-boost) (17), Extreme Gradient Boosting(XG-boost) (18), and generalized boosted regression modeling (GBM) (19), to further refine the valuable T cell exhaustion signature. In constructing the model, the output of the biomarkers from the machine learning models was intersected, followed by the utilization of multiple Cox regression to calculate the weight of each gene. The TEX-score formula is as follows:


TEX−Score=X1(coefficient of multi−COX of gene1)∗Y1(expression−level of gene1)+X2∗Y2+X3∗Y3

Based on the median value of the TEX-score, patients in the OS TARGETs cohort and the meta-cohort (formed by combining data from GSE21257 and GSE16091 using the R package “Combat”) were stratified into high and low TEX-score groups. Subsequently, Kaplan-Meier survival analysis and receiver operator characteristic curves (ROC) between these two groups were conducted using the “survminer”, “survival”, “rms”, and “timeROC” R packages.

### 2.7 Clinical characteristic and nomogram establishment

Uni-Cox and multi-Cox regression analyses were employed to assess the correlation and independence of the TEX-score in conjunction with clinical parameters in the meta-cohort. In order to delineate disparities between patient subgroups, a nomogram was developed. This nomogram is capable of accurately forecasting an individual’s probability of encountering an event in a clinical setting, incorporating independent clinical prognostic factors like age, gender, metastasis, and TEX-score. The performance of the nomogram in prognostic prediction was subsequently evaluated using calibration and ROC curves, validating its predictive capability for prognosis (20).

### 2.8 Evaluation of immune-related characteristics for the TEX-signature

The immune cell components in each sample were computed using the Tumor Immune Estimation Resource (TIMER), single sample gene set enrichment analysis (ssGSEA), and Microenvironment Cell Populations-counter (MCP-counter) algorithm (21). Additionally, the “ESTIMATE” package was utilized to estimate both stromal and immune scores, enabling the quantification of the Tumor Microenvironment (TME) in malignant tumors (22). The cancer immune cycle, encompassing seven distinct steps (TIP, hrbmu.edu.cn), as well as various immune indicators calculated by the “easier” package, were used to gauge the immune capacity of the TME (23). Furthermore, an examination was conducted into the expression levels of co-stimulatory, co-inhibitory, and HLA molecules. Parameters including T cell-inflamed gene expression profile (GEP), cytotoxic activity (CYT), and IFN-γ were computed in accordance with previously established methodologies (21, 24, 25). TME signatures, independently developed by Kobayashi, were gathered and computed utilizing Gene Set Variation Analysis (GSVA) (26).

### 2.9 Prediction of immunotherapy

The immunotherapy data was sourced from several datasets, namely GSE91061 (melanoma), GSE126044 (lung adenocarcinoma), Nathon (melanoma), and Mel-ucla (2016, metastatic-melanoma), which were utilized to forecast the response to immunotherapy (27, 28). Additionally, GSE79671 (glioblastoma) and GSE61676 (non-small cell lung cancer) were employed to assess the effectiveness of antivascular drugs within high and low TEX-score groups (29, 30). The TEX-score was calculated independently for each dataset. Subsequently, drug screening was conducted for patients with differing TEX-scores using the “oncopredict” package.

### 2.10 Quantitative real-time polymerase chain reaction

Ethical approval was obtained from the Medical Ethics Committee for tissue specimens acquired from the Third Xiangya Hospital of Central South University (Approval No. fast-23816). These specimens were stored at a temperature of -80°C. A total of three pairs of samples were collected from OS patients who underwent tumor resection, including tumor tissue and paratumor tissue. Total RNA from tissues was isolated using the TRIzol reagent by Thermo Fisher Scientific, based in Waltham, MA, USA. The cDNA was synthesized from 2μg of RNA utilizing the RevertAid™ First Strand cDNA Synthesis Kit (Thermo Fisher Scientific). Quantitative real-time polymerase chain reaction (qRT-PCR) was performed using SYBR Green Master Mix (Q111-02, Vazyme). The quantification of relative gene expression levels was conducted using the 2^-△△CT^ method. The primer sequences are shown in **Supplementary Table 1**.

### 2.11 Western blot analysis and Immunofluorescence

Protein samples were collected using RIPA buffer (Beyotime, China) and the protein concentration was determined using a bicinchoninic acid (BCA) assay kit (Chinese Biotechnology Company). A total of 20 μg of protein was separated by 12% SDS-PAGE and transferred onto PVDF membranes (Bio-Rad). After blocking with 5% non-fat milk at room temperature for one hour, the membranes were incubated overnight with antibodies diluted in antibody solutions against RAD23A (Immunoway), SAC3D1 (Immunoway), GAPDH (Immunoway), and PSIP1 (Proteintech). Following washing, the membranes were then incubated with ananti-rabbit IgG solution at room temperature for one hour, followed by additional washing and visualization. For histological analysis, the specimens were fixed in 4% paraformaldehyde after removal, and the fixed tissues were embedded in paraffin for sectioning and subsequent staining. The antibodies used for immunofluorescence staining were as follows: anti-human CD8 (Abcam); anti-human RAD23A (Immunoway); anti-human SAC3D1(Immunoway), anti-human PSIP1(Proteintech), Alexa 546-conjugated anti-rabbit IgG (Invitrogen). Cell nuclei were counterstained with DAPI (Sigma Aldrich). Imaging was performed using a Zeiss Axio Observer Z1 LSM 710 BiG confocal microscope (Carl Zeiss), and fluorescence images were captured using Zen 2012 software (Carl Zeiss). Images were pseudocolored for overlay, cropping, resizing, and enhancing contrast and brightness using Photoshop and Illustrator (Adobe Systems) or ImageJ (NIH).

### 2.12 Statistical analysis

The statistical analyses were performed using R (version 4.2.2) and RStudio. A prognostic model for OS was developed employing Combined LASSO regression, Boruta, survival-SVM, Cox-boost, XG-boost, and GBM. For survival analysis and assessing the diagnostic value of the TEX-signature, Kaplan-Meier curves and the Area Under the Curve (AUC) of the Receiver Operating Characteristic (ROC) were employed, respectively. In cases of normally distributed variables, significant quantitative differences between and among groups were determined using a two-tailed t-test or one-way ANOVA, as applicable. Conversely, for non-normally distributed variables, significant quantitative differences were assessed using a Wilcoxon test. A statistical P-value<0.05 was considered to be statistically significant.

## 3 Results

### 3.1 Single-cell analysis explored cell subtypes in OS

**Figure 1:**
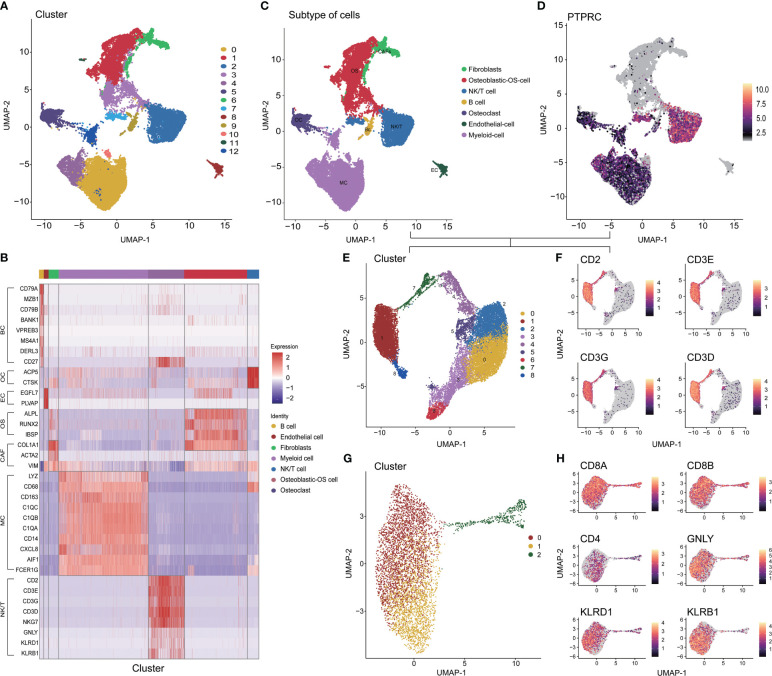
Different cell clustering in single cell sequencing data of osteosarcoma. **(A)** Identification 13 types of cells in single cell sequencing data. **(B)** Co-heatmap of marker genes for different cell types. **(C)** The 7 cell types were identified by marker genes. **(D, E)** Extraction, recombination and annotation of immune cells. **(F-H)** Screening of CD8+ T cell and NK/NKT cell cluster. OS: Osteoblastic-OS cell, EC: Endothelial cell, OC: Osteoclast, CAF: Fibroblasts, MC: Myeloid cell, NK/T: NK/NKT/T cell.

After controlling data quality and curating single-cell sequencing data from 6 OS patients, a total of 31,398 cells were screened and visualized through uniform manifold approximation and projection (**Supplementary Figures S1** and **2A**). The optimal number of cell populations was determined using the Seurat package, resulting in the differentiation of all cells into 13 distinct clusters (**Figure 1A**). Using the differential expression of genes between these 13 major clusters, combined with corrections for cell-specific cell markers for all subpopulations, an annotated classification of each cellular subpopulation within the osteosarcoma tumor microenvironment was performed. This included both immune (such as myeloid cells, NK/T cells, and B cells) and non-immune cells (such as osteoblastic OS cells, endothelial cells, OCs, and CAFs) (**Figure 1B, C** and **Supplementary Figures 2B-H**). After a comprehensive examination of the landscape and dynamics of immune cells, all groups of immune cells were re-grouped and annotated (**Figures 1D, E**). The NK/T cells underwent a similar process, while CD8^+^ T cells were specifically singled out for subsequent research (**Figures 1F-H**). Additionally, we observed a low expression level of CD4 within the T cell subpopulation (**Figure 1H**).

### 3.2 Analysis of CD8^+^ T cell site differentiation, cell population communication, and functional enrichment

**Figure 2:**
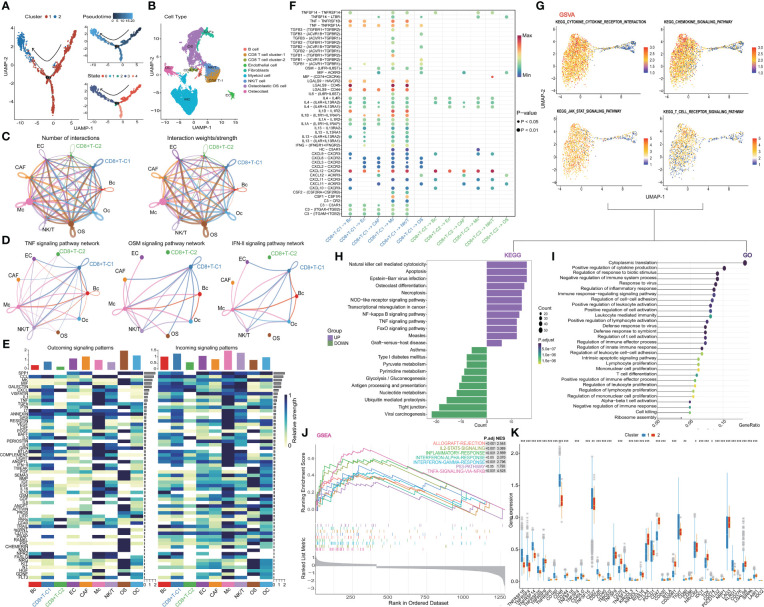
Further analysis of CD8+ T cell. **(A)** Trajectory plots showing different clusters in CD8+ T cells. **(B)** Rearrangement of cell types in TIME **(C)** The number and weights/strength of interactions in the cell-cell communication network. **(D)** Weight of classical inflammatory activation pathways. **(E)** Signal pattern and weight of the cytokine family **(F)** Visualization of the signal intensity of ligand interaction. **(G)** GSVA analysis of CD8+ T cells. **(H-J)** The outstanding enrichment of GO, KEGG, and GSEA terms in cluster 1. **(K)** Box plots comparing co-stimulatory and co-inhibitory molecules between two clusters. CD8+T-C1: CD8+T cell cluster 1, CD8+T-C2: CD8+t cell cluster 2.

The pseudo-time series analysis revealed the differentiation status among distinct clusters of CD8^+^ T cells and the rearrangement of cell types within the Tumor Microenvironment (TME) of OS (**Figures 2A, B**). Subsequently, we delved into the communication network among cell populations and found that CD8^+^ T cell cluster one exhibited a more active interaction status and weight compared to CD8^+^ T cell cluster two (**Figure 2C**). Classical inflammatory activation pathways such as TNF, OSM, and IFN-II signaling pathways displayed heightened activity in CD8^+^ T cell cluster one and Myeloid cells. Similarly, we investigated the signaling pattern and weight of cytokine families like IL-1, IL-2, IL-4, and IL-6 pathways, along with signaling pathways including TGF-beta, CCL, CD40, complement, and TRAIL in clusters (**Figure 2D, E** and **Supplementary Figure 3A**). In summary, CD8^+^ T cell cluster one demonstrated more pronounced advantages than cluster two across most inflammatory and immune activation pathways. Further visualization of the ligand interaction signal intensity revealed that the ligand interaction between CD8^+^ T cell cluster two and others was relatively attenuated in comparison to CD8^+^ T cell cluster one (**Figure 2F**). Furthermore, gene enrichment analysis was conducted between the two subtypes of CD8^+^ T cells to validate our hypothesis. The GSVA results of KEGG terms demonstrated a strong association of cluster one with cytokine, JAK-STAT, and T cell receptor signaling pathways (**Figure 2G**). Additionally, KEGG analysis was carried out by evaluating the up- and down-regulated differentially expressed genes in subgroup one of CD8^+^ T cells. It revealed that major pathways in cytotoxicity mediated by NK cells, necroptosis, TNF, and NOD signaling were positively enriched (**Figure 2H**). As for GO terms, a plethora of immune processes exhibited significant enrichment in cluster one, including inflammatory response, lymphocyte migration, proliferation and activation, as well as T cell differentiation and activation regulation (**Figure 2I**). Furthermore, the hallmark pathways of GSEA in cluster one indicated that molecules and pathways associated with immune function were highly activated (**Figure 2J**). In contrast, cluster two exhibited greater enrichment in metabolism-related pathways and lacked immune activation in GO, KEGG, and GSEA analyses (**Figure 2H**, **Supplementary Figures 3B, C**). Finally, the overall expression status of co-stimulatory and co-inhibitory molecules was compared between the two clusters (**Figure 2K**). Combining these findings with our previous results, we propose a process of functional exhaustion in the differentiation of CD8^+^ T cells between the two subsets.

### 3.3 Exploring the genetic changes associated with exhaustion phenotype

**Figure 3:**
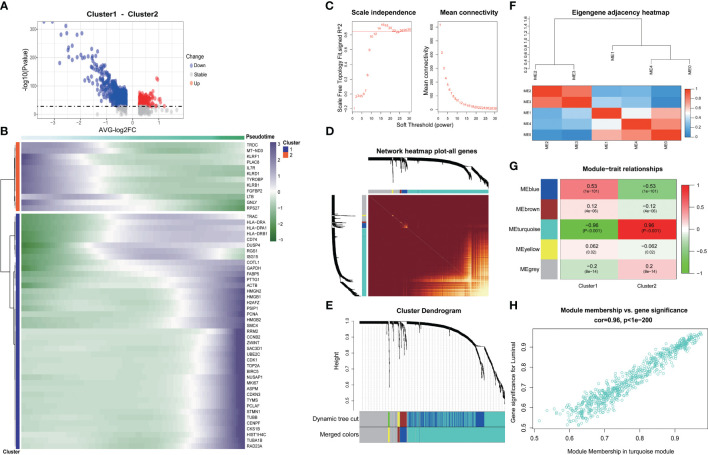
Exploring the genetic changes associated with exhaustion phenotype. **(A)** Volcano map of differentially expressed genes in CD8+ T cell clusters. **(B)** Heat map of differentially expressed genes in CD8+ T cell clusters. **(C)** Scale independence and average connectivity. **(D)** Network heatmap plot of all genes. **(E)** Cluster dendrogram. **(F)** Eigengene adjacency heatmap. **(G)** Heatmap of module–trait correlations. **(H)** Correlation between gene significance and module membership.

We delved into the core gene-level alterations within the differentiation trajectory of CD8^+^ T cell subpopulations and found that there were noteworthy disparities in core genes between different clusters (**Figures 3A, B**). We utilized the hd-WGCNA algorithm to compute the gene expression profiles of the two CD8^+^ T cell subsets, and then categorized the core genes between these subsets into distinct gene modules to identify the core gene sets. Finally, we verified the correlation between genes and modules in the network. By setting β to 14, we achieved an R-squared value of 0.85, which established a scale-free network (**Figure 3C**). The genes were segregated into respective modules via hierarchical clustering, and a gene similarity heatmap was generated based on the topological overlap matrix (**Figures 3D-F**). The core genes were predominantly concentrated in the turquoise module, with a remarkably high correlation of 96% (**Figure 3G**). Further analysis revealed a strong correlation between genes within the block and the block (**Figure 3H**).

### 3.4 Machine learning to build TEX-signature

**Figure 4:**
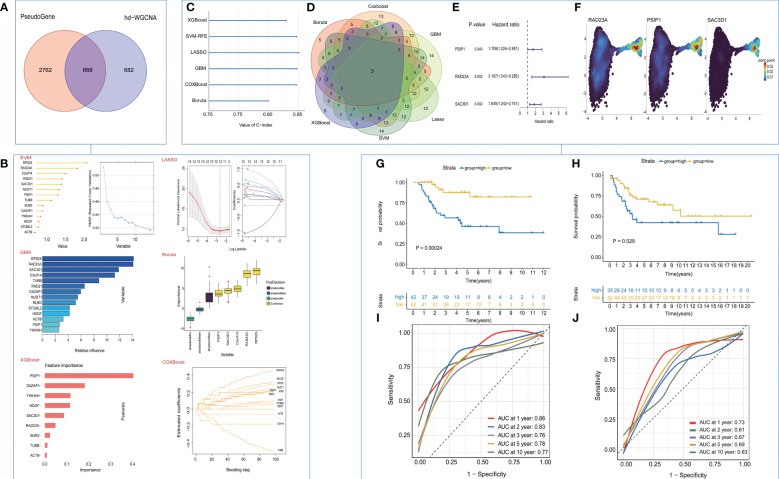
Construction and validation of the TEX-Signature. **(A)** Venn diagram of exhausted genes and core genes of the turquoise module. **(B)** machine learning for valuable models. **(C)** The C-index of various algorithms. **(D)** Venn plot showing the intersection of valuable TEX-genes based on six machine learning algorithms. **(E)** coefficient of three target genes calculated by multivariate analysis **(F)** Density plots of gene expression intensity for target genes. **(G)** Kaplan-Meier survival curve of OS between patients with a relative high score of TEX-Signature and a low score of TEX-Signature in the TARGETs cohort. **(H)** Kaplan-Meier survival curve of OS between patients with a high score of TEX-Signature and a low score of TEX-Signature in the meta-cohort. **(I)** Time-dependent ROC curve at 1, 2, 3, 5, and 10 years in the TARGETs cohort. **(J)** Time-dependent ROC curve at 1, 2, 3, 5, and 10 years in the meta-cohort.

In our initial investigation into the biomarkers of T cell exhaustion, which have prognostic significance for patients with OS, we identified 668 genes that were commonly present in both cohorts (**Figure 4A**). Following univariate analysis of these exhausted core genes, feature selection was performed using six machine learning algorithms, including LASSO, XGboost, GBM, Boruta, CoxBoost, and survival-SVM (**Figure 4B**, **Supplementary Figure 4A**). The C-index values for all the algorithms exceeded 0.8, indicating the strong performance of each model (**Figure 4C**). Subsequently, we selected the intersection of the biomarkers obtained from the machine learning model to construct a refined model (**Figure 4D**). Three target genes, RAD23A, SAC3D1, and PSIP1, were screened along with their corresponding coefficients calculated through multivariate analysis (**Figure 4E**). Expression of target genes between the two cell clusters was also visualized (**Figure 4F**). Moreover, we defined the TEX-score as the sum of the product of the expression values and the correlation coefficients of these three genes separately. Comparisons of the prognostic status of patients with high TEX-scores against those with low TEX-scores revealed that in both the TARGETs cohort and the meta-cohort, patients with high TEX-scores exhibited worse clinical outcomes, while those with low TEX-scores demonstrated better outcomes (**Figures 4G, H**). Additionally, the area under the ROC curve demonstrated the excellent diagnostic efficacy and predictive ability of the model at 1, 2, 3, 5, and 10 years in both the TARGETs cohort and the meta-cohort (**Figures 4I, J**).

### 3.5 Construction of a nomogram and clinical characteristic subgroup analysis

**Figure 5:**
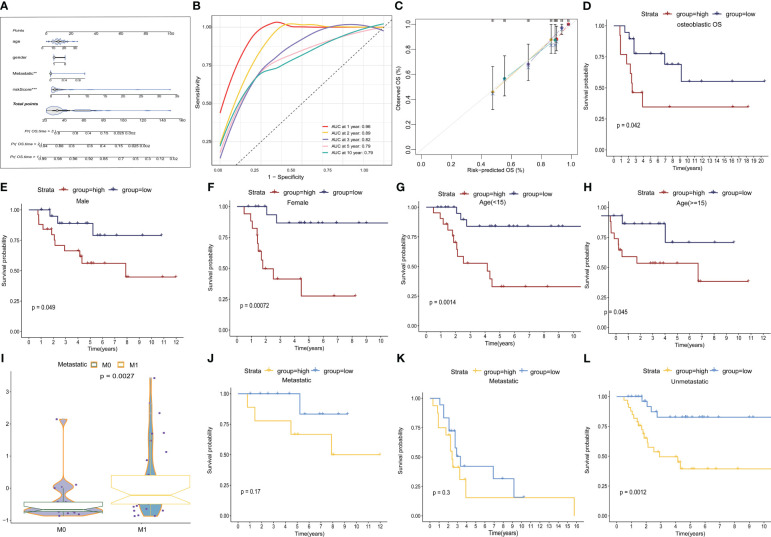
Construction of a nomogram and subgroup analysis. **(A)** Nomogram based on gender, age, metastasis and the TEX-score. **(B)** ROC curves for predicting 1-, 2-, 3-, 5- and 10-year survival in the TARGETs database. **(C)** The calibration plot for the probability of 1-, 2-, 3- and 5- year overall survival of OS patients. **(D-H)** Kaplan–Meier survival analysis for OS patients with diverse clinical characteristics of osteosarcoma type **(D)**, gender **(E, F)**, age **(G, H)** in TARGETs cohort. **(I)** Boxplot of TEX scores between metastatic and non-metastatic patients in the GSE21257 dataset **(J-L)** Kaplan-Meier survival analysis of metastatic and non-metastatic patients in the TARGETs **(J)** and the GSE21257 dataset **(K-L)**.

We developed a nomogram that incorporated age, gender, metastasis, and TEX-scores for clinical prediction (**Figure 5A**, **Supplementary Figures 4B–E**). The AUC values for 1-, 2-, 3-, 5-, and 10-year Overall Survival (OS) for the nomogram were 0.96, 0.89, 0.82, 0.79, and 0.79, respectively, indicating that our model exhibited strong and consistent predictive capability (**Figure 5B**). Furthermore, the calibration plots illustrated the level of agreement between the predicted OS and the actual OS (**Figure 5C**). To further underscore the predictive potential of the TEX-signature, we conducted subgroup analyses based on available clinical features in the TARGETs and GSE21257 databases. The signature demonstrated accurate and robust performance across these subgroups. According to Kaplan-Meier survival analysis, the low TEX-score group consistently exhibited a superior prognosis compared to the high TEX-score group within subgroups stratified by OS type, gender, age, or metastasis (**Figures 5D–L** and **Supplementary Figures 4F–J**). In addition, there is a tendency for the TEX-score to decrease with age, suggesting to some extent that they may be generalizable (**Supplementary Figures 4K-L**).

### 3.6 Immune characteristics related to the TEX-signature

**Figure 6:**
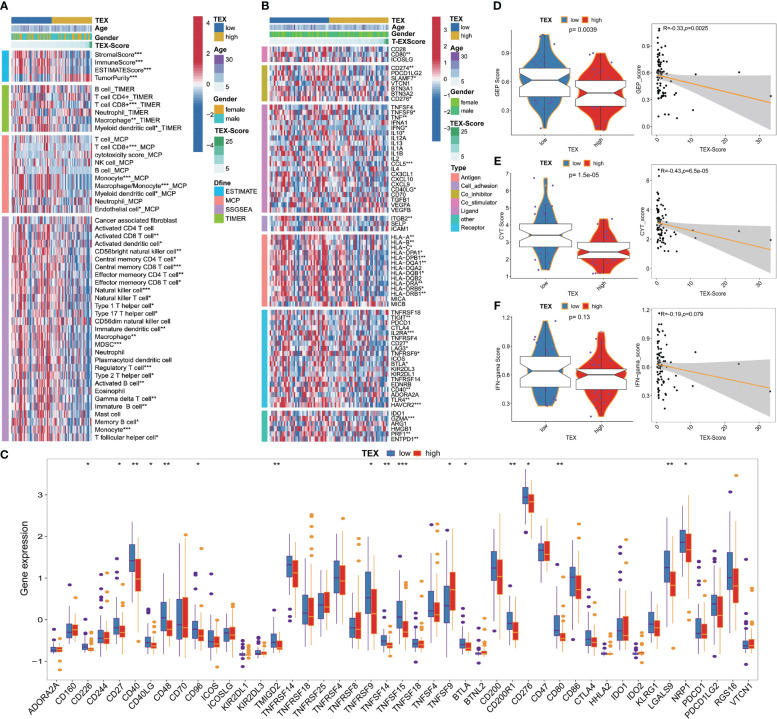
Immune-related characteristics of TEX-signature in TARGETs and Meta-cohort. **(A)** Heatmap showing the correlation between TEX**-**score and immune infiltrating. **(B)** Heatmap showing the correlation between TEX**-**score and immune modulators. **(C)** Box plots comparing the expression status of all immune checkpoint molecules between low and high TEX-score groups. **(D-F)** Boxplot and scatter plot displaying the levels of GEP, CYT, and IFN-γ between low and high TEX-score groups. *p<0.05, **p<0.01, ***p<0.001.

We investigated the association between TEX-signature and immune cell infiltration as well as immunomodulators in both the TARGETs cohort and the meta-cohort to evaluate the impact of TEX-signature in OS. Patients with high TEX-scores displayed a strong correlation with tumor purity, whereas the low TEX-score group exhibited a more favorable immune microenvironment and matrix score (**Figure 6A** and **Supplementary Figure 5A**). Moreover, we discovered that the low TEX-score group had positive associations with immune cells such as CD8^+^ T cells, macrophages, natural killer cells (NK cells), NK T cells, B cells, and central and effector memory T cells, all of which play important roles in positive immune regulation and immune-mediated killing utilizing multiple algorithms including ESTIMATE, TIMER, MCP-counter, and ssGSEA (**Figure 6A**, **Supplementary Figures 5B–D**). However, myeloid-derived suppressor cells (MDSC) were also more enriched in the low TEX-score group (**Figure 6A** and **Supplementary Figures 5B-D**). Additionally, the TEX-score exhibited negative correlations with most immune modulators, classified as antigen presentation, co-stimulatory, co-inhibitory, receptor, and others in the TARGETs cohort. The expression status of all immune checkpoint molecules was also depicted (**Figures 6B, C**). Furthermore, we confirmed the expression levels of co-stimulatory, co-inhibitory, and HLA molecules in the meta-cohort (**Supplementary Figures 5E, F**). Finally, we explored several immunotherapy indices in both the TARGETs cohort and meta-cohort. High levels of GEP, CYT, and IFN-γ were significantly associated with a low TEX-score, all of which are determinants of a potentially improved immunotherapy response (**Figures 6D-F** and **Supplementary Figures 5G-I**). Interestingly, there was no statistically significant difference in IFN in the TARGETs cohort, although our validation in the meta-cohort indicated that the results were still meaningful. Our results showed a clear intrinsic correlation between the immune microenvironment and TEX-scores, with the low TEX-score patients having an “immune-heat response phenotype” reflecting a better immunotherapeutic potential, whereas the high TEX-score group showed an “immune-poor state”.

### 3.7 Potential biological process related to TEX-signature

**Figure 7:**
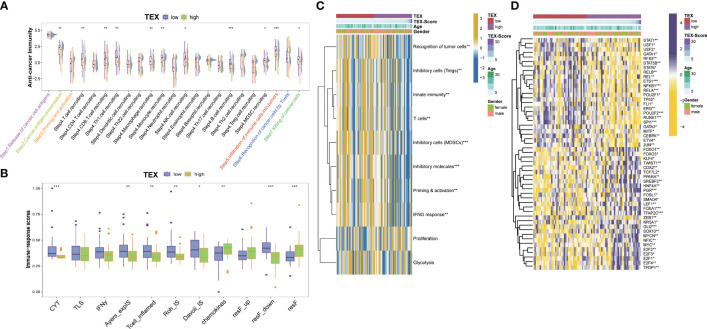
Potential biological process of the TEX-signature. **(A)** Boxplot showing the differences in the cancer immunity cycle between low and high TEX-score groups. **(B)** The differences in immune-related indexes collected from the “easier” package in groups. **(C)** Heatmap showing the correlation between TEX-score and immune level developed by Kobayashi. **(D)** Heatmap showing the correlation between the TEX-score and transcription factors. *p<0.05, **p<0.01, ***p<0.001.

We examined the immunity cycle of cancer to elucidate the relationship between immune processes and TEX-score across the entire dataset, several steps of the immune cycle were found to be more activated in the low TEX-score group in our study. They included cancer antigen presentation, recruitment of T cells, CD8^+^ T cells, Th1 cells, NK cells, macrophages, B cells, infiltration of immune cells into tumors, and killing of cancer cells (**Figure 7A**). Moreover, we gathered various indices, including the T cell inflammatory microenvironment signature (T-cell-inflamed), which is based on the combined potential of IFN-γ and T-cell-associated inflammatory genes in predicting the response to PD-1 blockade, as well as the immunological characteristics of Roh (Roh-IS) associated with immune activation related to tumor rejection, and the immunological characteristics of Davoli (Davoli-IS), defined by the expression of cytotoxic CD8^+^ T cell, NK cell markers, and immuno-expanded label (Ayers-expIS), which is produced by genes highly associated with IFN-γ signature genes. All of these scores were highly significant in the low TEX-score group (**Figure 7B**). The immune resistance program (resF-down, resF-up, and resF) represents the efficacy of immune resistance in the tumor microenvironment, with patients in the high TEX-score group exhibiting stronger immune resistance (resF, resF-up), while lower levels of immune resistance (resF-down) were present in the low TEX-score group (**Figure 7B**). Furthermore, we examined the signatures developed by Kobayashi in the TARGETs cohort, where a low TEX-score was associated with higher levels of recognition of tumor cells, innate immunity, T cells, IFN-γ response, Tregs, and MDSCs, while proliferation levels were positively correlated with TEX-scores (**Figure 7C**). Additionally, we found that transcription factors associated with inflammation and tumor suppression, such as USF1, USF2, RFX5, TP53, ETS1, SPI1, GATA, and STAT1, were highly expressed in the low TEX-score group. Factors that play a bidirectional role in proliferation and immunity, including NF-κB, STAT5B, and STAT6, were also highly expressed in the low TEX-score group. Other major potential tumor growth factors, such as POU2F2, RUNX1, ERG, REL, and JUN, were also relatively increased in the low TEX-score group. Except for FOXO1, KLF4, and SMAD4, the highly expressed transcription factors promoted OS proliferation, metastasis, and drug resistance in the high TEX-score group, including the E2F family, MYC, TFDP1, ZEB1, TFAP2C, LEF1, FOSL1, TCF7L2, TWIST1, GLI2, and FOXO3 (**Figure 7D**).

### 3.8 Function enrichment and metabolism of TEX-signature

**Figure 8:**
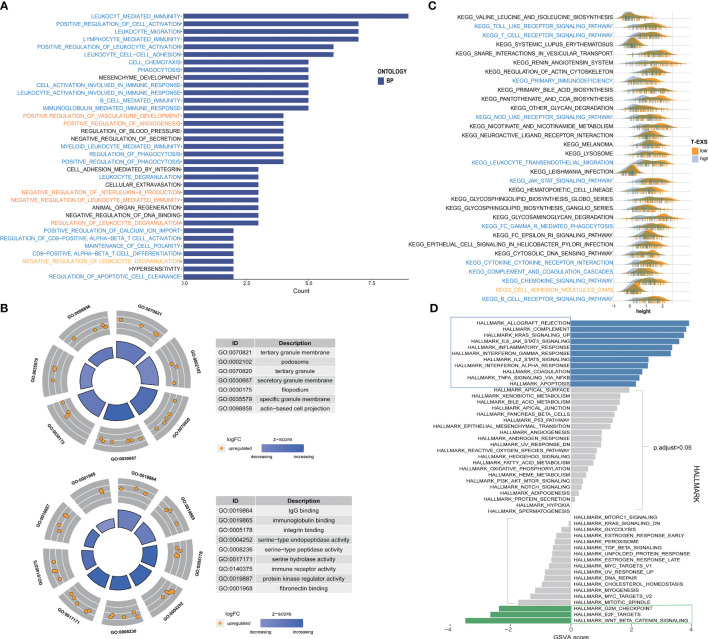
Function enrichment and metabolism of the TEX-signature. **(A)** Biological process of GO analysis in the low TEX-score group. **(B)** Cell component and molecular function of GO analysis in the low TEX-score group. **(C)** KEGG analysis in the low TEX-score group. **(D)** GSVA analysis of low and high TEX-score groups.

We examined the similarities and differences between TEX-score subgroups at the level of specific biological functions and pathways. The GO analysis of biological processes primarily encompassed positive reactions of leukocytes, such as immune migration, adhesion, activation, and phagocytosis in the low TEX-score group. This specifically included the activation and differentiation of CD8^+^ T cells, B cells, and myeloid cell-mediated immunity (**Figure 8A**). The cellular components identified in the GO analysis were associated with membrane and filopodium components. Molecular functions included IgG binding, immunoglobulin binding, immune receptor activity, and serine-type peptidase activity (**Figure 8B**). The GO analysis results for the high TEX-score group were also notable. There was enrichment related to ion channels, both voltage-dependent and independent in biological processes. Terms like transporter complex and channel complex were enriched for cellular components. Bone morphogenesis and ossification were significantly enriched in terms of molecular functions (**Supplementary Figure 6A**). Additionally, we conducted a KEGG analysis that showed a significant enrichment in the low TEX-score group. We visually compared the enrichment status of the corresponding pathways in the two subgroups. Pathways including the Toll-like receptor, T cell receptor, NOD-like receptor, leukocyte trans-endothelial migration, Fc-γ receptor-mediated phagocytosis, cytokine-cytokine receptor interaction, chemokine signaling pathway, and B cell receptor, all of which were involved in immune processes, were significantly associated with the low TEX-score group (**Figure 8C**). Furthermore, numerous Hallmark signaling pathways of GSVA correlated with the low TEX-score, included complement, IL6-JAK-STAT3, and IL2-STAT5 signaling pathway, inflammatory response, IFN-α and IFN-γ response, and TNFα-NF-kB signaling pathway. As for the high TEX-score group, the results were consistent with what we obtained previously (**Figure 8D**).

### 3.9 Predictive efficacy of TEX-signature for therapy

**Figure 9:**
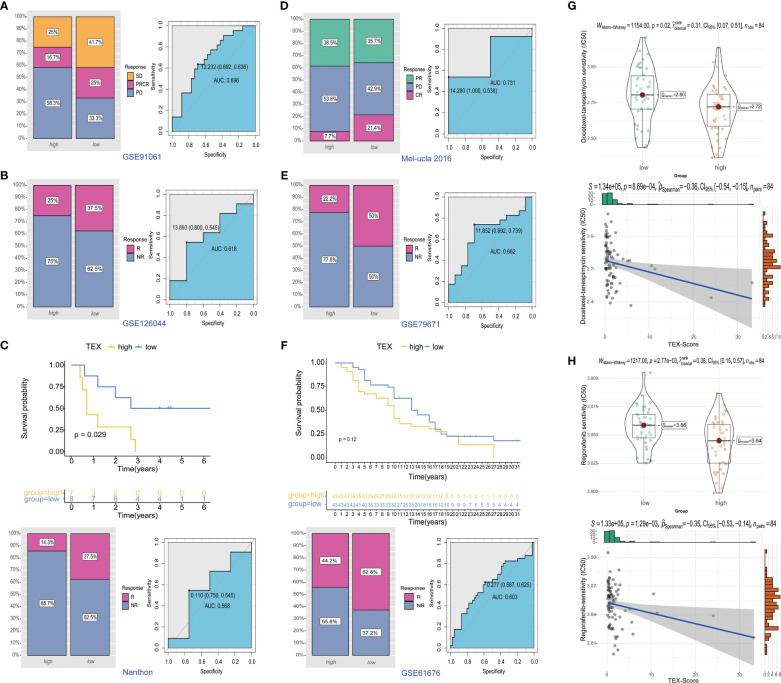
Predictive value of TEX-signature in therapy response. **(A-D)** Distribution of immunotherapy responses and ROC curves in the GSE91061 cohort, GSE126044 cohort, Nanthon dataset and Mel-ucla dataset based on the TEX-signature **(E, F)** Distribution of anti-angiogenic therapy responses and the ROC curve between low and high TEX-score groups in the GSE79617 and GSE61676 dataset. **(G, H)** The sensitivity of docetaxel-tanespimycin and regorafenib in two groups.

To further investigate the potential value of the TEX-signature in therapy response, we proceeded to validate its efficacy in multiple published therapy datasets. The predictive capacity of the TEX-signature was well-evidenced by Disease Control Rate (DCR) in the context of immunotherapy. Patients with low TEX-scores in the GSE91061 cohort exhibited significantly improved DCR compared to patients with high TEX-scores, and the ROC curve confirmed the robustness of the TEX-score in predicting therapy response (**Figure 9A**). Similarly, patients with low TEX-scores in the GSE126044 dataset demonstrated a higher likelihood of responding positively to immunotherapy (**Figure 9B**). Patients with low TEX-scores in the Nanthon dataset experienced extended survival times and were more inclined to respond to immunotherapy (**Figure 9C**). Patients with low TEX-scores in the Mel-ucla dataset exhibited a superior DCR (**Figure 9D**). Turning to anti-angiogenic therapy, patients with low TEX-scores in the GSE79671 dataset were more prone to positive responses to anti-angiogenic therapy (**Figure 9E**). Likewise, in the GSE61676 dataset, patients with low TEX-scores demonstrated prolonged survival times and were more likely to respond favorably to anti-angiogenic therapy (**Figure 9F**). In addition, we verified the predictive value of the TEX-signature for chemotherapies. As shown, the TEX-score showed a significant correlation with major chemotherapeutic agents, including docetaxel-tanespimycin, regorafenib, sorafenib, topotecan, pazopanib, and paclitaxel. Patients with high TEX-scores appeared to be more likely to respond positively to chemotherapies (**Figures 9G, H** and **Supplementary Figures 6B-E**). Taken together, our research revealed that patients with low TEX-scores could potentially benefit more from certain treatment options.

### 3.10 The validation of TEX-related gene expression

**Figure 10:**
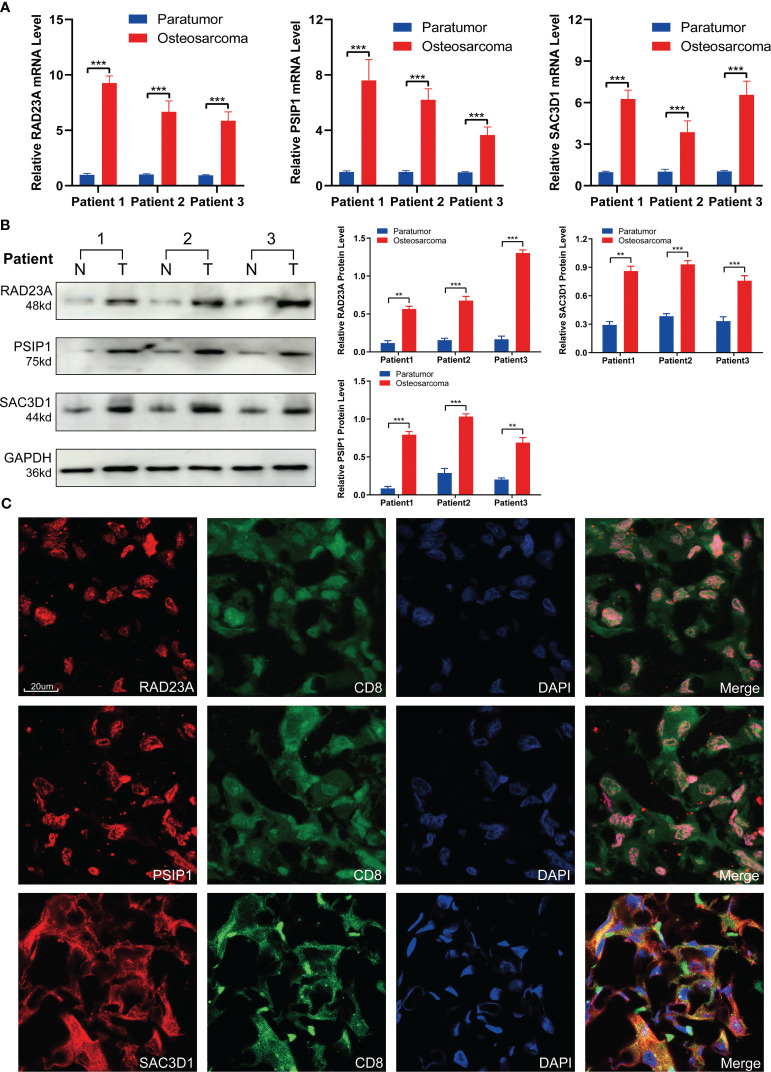
Validation of TEX-related gene expression. **(A)** QRT-PCR analysis and **(B)** Western blot analysis of RAD23A, PSIP1, and SAC3D1. **(C)** Immunofluorescence co-localization of CD8 and TEX-related genes was performed under 40x magnification. *P < 0.05, **P < 0.01, ***P < 0.001.

To validate the expression patterns of TEX-related genes in osteosarcoma (OS) patients, we performed RT-PCR and Western Blot analyses on tumor tissues and adjacent non-tumor tissues from three patients. The results showed that, compared to adjacent non-tumor tissues, the expression of RAD23A, SAC3D1, and PSIP1 was significantly upregulated in OS tissues (**Figures 10A, B**). Additionally, we characterized the localization expression of the three target genes in CD8 T cells using immunofluorescence in OS tissue (**Figure 10C**). Therefore, we propose that dysregulated expression of these genes may lead to T-cell exhaustion and promote OS progression.

## 4 Discussion

Chemotherapy and surgical resection have long been the mainstay of treatment for OS (31). Unfortunately, there has been limited advancement in the treatment of OS over the last three decades, particularly in contrast to the notable progress made in developing novel therapies for other types of cancer (32). This stagnation in innovation has regrettably not translated into improved survival rates for patients dealing with OS. The need for further research and breakthroughs in treatment options for OS remains paramount.

Immunotherapy, an emerging therapeutic approach, has made significant strides in treating various cancer types. However, its impact on OS has been relatively limited (33). Effectively reshaping the immunosuppressive tumor microenvironment is crucial for the success of immunotherapy (34). Nevertheless, the intricate interplay of factors including the complex immune microenvironment, tumor heterogeneity, and individual variations in OS poses formidable challenges to harnessing the full potential of immune-based treatments (35). The core of immunotherapy is the activated T cells, particularly CD8^+^ T cells, whose functional state closely correlates with immune response effectiveness. However, CD8^+^ T cells are often altered or exhausted due to prolonged exposure to high levels of persistent antigen and inflammatory stimuli during tumor progression. These exhausted T cells lose their ability to eliminate tumor cells (11, 36). Immune checkpoint inhibitors such as anti-PD-1 antibodies and anti-CTLA-4 antibodies, regulatory cytokines, and metabolic reprogramming targeting the tumor microenvironment work to reverse the exhausted T cell states, restore their functionality, and reactivate immune responses. Previous studies have indicated that the number of tumor-infiltrating lymphocytes (TIL) is significantly higher in OS compared to other sarcomas (37), which suggests that immune checkpoint inhibitors may be able to leverage the abundance of TIL in OS, offering hope for immunotherapy in this context. However, the relationship between T cell exhaustion and OS remains inadequately understood.

CD8^+^ T cells, originating from CD34 hematopoietic stem cells located in the bone marrow, can be activated by endogenous antigenic peptides presented in MHC class I molecules, thereby exerting anti-tumor immunity (38). When the functionality of CD8^+^ T cells is compromised, the body’s anti-tumor immune capacity diminishes, elevating the risk of tumor growth and cancer metastasis (39). Through an exploration of the molecular and functional attributes of distinct CD8^+^ T cell subgroups in OS, we observed indications of functional exhaustion within the tumor immune microenvironment. In contrast to relatively exhausted CD8^+^ T cells, their more active counterparts demonstrated heightened engagement in cellular interactions, with most immune-related pathways exhibiting elevated activity. These pathways encompassed inflammatory activation pathways, TNF family members, complement C3, cytokine family, immune response, cell-cell adhesion, necroptosis, and T cell activation. The relatively exhausted cell subgroup exhibited heightened expression of markers like LAG-3, TOX, CTLA-4, aligning with prior studies elucidating mechanisms associated with CD8^+^ T cell exhaustion (40). This expression profile may potentially impact the immune response and prognosis of OS patients. These findings inspire us to further refine and scrutinize exhaustion models, seeking additional insights to advance immunotherapy for OS.

Following the application of six machine learning algorithms, we identified RAD23A, SAC3D1, and PSIP1 as genes associated with T cell exhaustion, forming the basis for an OS prognostic model. RAD23A, also known as RAD23 or HR23A, is involved in nucleotide excision repair and the regulation of intracellular protein degradation (41). Previous pan-carcinoma analyses have indicated a significant positive correlation of RAD23A in various cancers (42). It participate in processes such as nuclear translocation of AIF during cell death induction and enhances resistance to chemical agents by modulating autophagic response (43). RAD23A may mediate T cell exhaustion through diverse pathways and is recognized as an immune function biomarker, substantiating its inclusion in the prognostic model (44). SAC3D1, or SHD1, is implicated in centrosome duplication and mitotic progression, potentially mediating cell cycle regulation via centrosome amplification (45). SAC3D1 is involved in immune response, as well as its association with metabolism-related signaling pathways, positions it as a key player in T cell exhaustion and provides valuable insights for prognosis and immunotherapy effectiveness in various cancers (46, 47). PSIP1, also known as LEDGF/p75, participates in various biological processes and plays a significant role in lens epithelium differentiation into fiber cell terminals (48). The precise influence of these genes on the occurrence and progression of T cell exhaustion, particularly in relation to CD8^+^ T cell exhaustion in OS, deserves further exploration. Indeed, understanding the intricate interplay between the tumor immune microenvironment and T cell exhaustion is crucial for unraveling the complexities of cancer progression and devising effective therapeutic strategies (49, 50). The observations made in this study regarding immune cell infiltration and immune checkpoints between individuals with high and low TEX-scores shed light on potential avenues for enhancing immune efficacy. The heightened presence of CD8+ cells, macrophages, NK cells, NK T cells, B cells, and monocyte cells in the low TEX-score group signifies a more active immune response, which aligns with the notion of reduced T cell exhaustion. Moreover, the association of immune pathways with TEX-score subgroups provides valuable insights into the potential effectiveness of immunotherapy in OS. The activation of the WNT/β-catenin signaling pathway in the high TEX-score group is particularly noteworthy, as this pathway is known to play a crucial role in T cell differentiation and effector function (51). Previous studies have demonstrated that the activation of WNT/β-catenin signaling can suppress the effector functions of CD8+ T cells, further emphasizing its relevance in the context of T cell exhaustion (52). The expression of immune checkpoint molecules can originate from various cell types, including tumor cells, regulatory T cells (Treg), fibroblasts, or their extracellular vesicles. In the low-TEX group, co-stimulatory molecules such as CD40/CD40LG and CD96 were relatively upregulated, accompanied by elevated levels of TNF-α, GZMA, and IFN-α/IFN-γ. Meanwhile, we observed a relative upregulation of some inhibitory immune checkpoints, this may be attributed to heightened antigen presentation stimulation due to high HLA expression and a tumor’s self-protective effect induced by sustained inflammatory responses. The upregulation of CD274, IDO1, etc. on the tumor surface by T cell activation and IFN-α/IFN-γ stimulation has been demonstrated (53, 54). Furthermore, the Meta-dataset’s high-TEX group showed increased expression of TOX and VCTN1, and the relationship between immune checkpoint regulation and immune infiltration was more intricate than we had first thought. Patients with elevated immune checkpoint levels may also exhibit higher levels of immune activation, and this group of patients may experience better clinical benefit from combination immunotherapy (55). This highlights the potential for interventions aimed at reversing the state of immune exhaustion in the tumor microenvironment, a development that could have far-reaching implications for cancer therapy. Reassuringly, recent clinical successes in reversing T cell exhaustion underscore the promising potential of such approaches (56).

As we know, T cell exhaustion is a prolonged and persistent process characterized by the upregulation of various immune inhibitory factors and impaired functionality, such as compromised release of IFN-γ and granzymes, within the tumor immune microenvironment (TIME) under inflammatory stimuli. Despite being the mainstay of immunotherapy, classical immune inhibitors like Anti-PD1 and Anti-CTLA4, represented by immune checkpoint blockade (ICB), unfortunately, fail to provide long-term benefits for a significant proportion of patients (57). The restoration of exhausted T cell functions is often limited, and they can rapidly revert to their pre-treatment state. Current research has identified CD8+ and Th1-type T cell markers, including IFN-γ, PRF1, and TAP1, to be significantly correlated with patients’ responses to immunotherapy (58). Additionally, scholars have found that early PD-1 blockade combined with CAR-T therapy can achieve better prognosis improvement (59). Therefore, for patients with relatively low tumor heterogeneity, high immune infiltration, and limited exhaustion, immunotherapy may attain better long-term efficacy (60). In our research cohort, besides the significant correlation of important indicators such as GEP, IFN-γ, and CYT with low exhaustion levels, the consistent performance of scores like Roh-is, Davoli-is, and RIR further supports our hypothesis, affirming the favorable prognosis of low TEX and providing support for our hypothesis. The validation of this model in multiple therapy datasets across different tumor types further strengthens its predictive efficiency. The findings regarding the sensitivity of patients to anti-angiogenic drugs and conventional chemotherapy drugs for OS highlight the potential clinical utility of the TEX-signature in guiding treatment decisions. However, it’s important to acknowledge the need for further validation and clinical implementation. This study sets a promising foundation for future research and potential advancements in the treatment of OS.

It should be mentioned that there are a few of restrictions. Firstly, due to tumor heterogeneity and limited sample size, the study’s findings are based on single-cell sequencing data from a relatively small sample number, which may not fully capture the heterogeneity present in osteosarcoma. Further validation in larger cohorts would provide more robust and generalizable results. Second, while the study identifies core genes in the TEX-signature, further molecular experiments are necessary to elucidate the functional roles of these genes and understand the underlying molecular mechanisms of CD8^+^ T cell exhaustion. Finally, the study did not specifically address the prediction of metastasis in osteosarcoma. It’s important to acknowledge that the model’s performance in this regard remains uncertain, because we were unable to get reliable metastasis-related data.

In conclusion, our study aims to analyze the immune microenvironment and tumor heterogeneity in OS using single-cell sequencing data, identifying distinct differentiation trajectories of CD8^+^ T cells in different individuals, and conducting a thorough evaluation of CD8^+^ T cells, which holds promise in shedding light on new avenues for OS immunotherapy.

## Data availability statement

The datasets presented in this study can be found in online repositories. The names of the repository/repositories and accession number(s) can be found in the article/**Supplementary Material**.

## Ethics statement

The animal study was approved by the Medical Ethics Committee of the Third Xiangya Hospital of Central South University. The study was conducted in accordance with the local legislation and institutional requirements.

## Author contributions

QF: Conceptualization, Funding acquisition, Investigation, Writing – original draft, Data curation, Validation, Visualization. YW: Funding acquisition, Software, Conceptualization, Data curation, Investigation, Project administration, Visualization, Writing – original draft. JC: Data curation, Formal analysis, Methodology, Supervision, Writing – review & editing. BP:Methodology, Funding acquisition, Investigation, Validation, Writing – original draft. XZ: Validation, Funding acquisition, Writing – review & editing. RL: Investigation, Writing – original draft, Conceptualization, Data curation, Formal analysis. YD: Funding acquisition, Resources, Software, Supervision, Writing – review & editing.

## Supplementary material

Supplementary Figure 1 **(A)** Density plot of transcript counts detected in each sample as a proportion of cell number. **(B)** Total number of genes detected in each sample. **(C)** Distribution of the percentage of mitochondrial genes in each sample. **(D)** Correlation analysis of total transcript number with mitochondrial genes, total gene number and PCA clustering analysis of all cells.

Supplementary Figure 2 **(A)** Two-dimensional visualization of cell distribution across samples. **(B-H)** The “umap” visualization of cell subpopulation-specific marker expression levels.

Supplementary Figure 3 **(A)** Communication networks among cell populations of multiple signaling pathways in cluster 2. **(B)** The enrichment of GO and GSEA terms in cluster 2.

Supplementary Figure 4 **(A)** Risk coefficients for potential TEX-related genes calculated by univariate analysis. **(B-E)** The risk correlation analysis for each clinical characteristic and TEX-score in **(B-C)** The TARGETs and **(D-E)** the GSE21257 dataset. **(F)** Comparison of TEX scores of metastatic patients in the TARGETs cohort. **(G-J)** Kaplan–Meier survival analysis for OS patients with diverse clinical characteristics of age **(G, H)**, and gender **(I, J)** in the GSE21257 dataset. **(K-L)** Distribution of TEX scores for all patients by age.

Supplementary Figure 5 **(A-C)** Immune characteristics related to the TEX-signature. **(D)** Heatmap showing the correlation between TEX-score and immune infiltrating. **(E-F)** The expression status of immune checkpoint and antigen-presenting molecules between low and high TEX-score groups. **(G-I)** The levels of CYT, IFN-γ and GEP between low and high TEX-score groups.

Supplementary Figure 6 **(A)** Function enrichment and metabolism of the high TEX-score. **(B-E)** The sensitivity of Sorafenib, Topotecan, Pazopanib, and Paclitaxel between low and high TEX-score.
