# Supplementary figures and images for: Single-cell RNA-seq reveals T cell exhaustion and immune response landscape in osteosarcoma
Source: Front Immunol. 2024 Apr 2;15:1362970. doi: 10.3389/fimmu.2024.1362970 (PMC11018946; doi:10.3389/fimmu.2024.1362970)

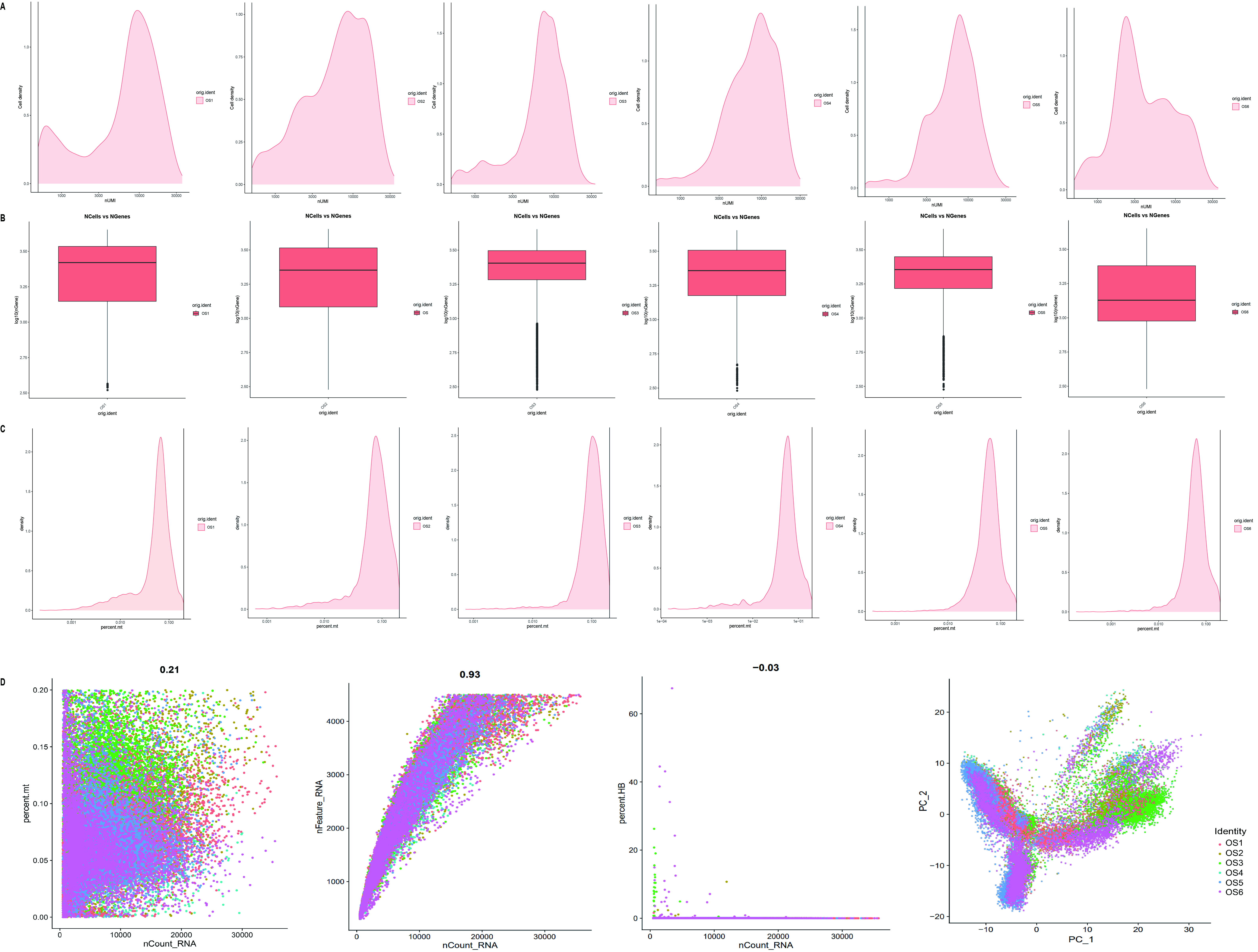

Supplement: Supplementary Figure 1 — (A) Density plot of transcript counts detected in each sample as a proportion of cell number. (B) Total number of genes detected in each sample. (C) Distribution of the percentage of mitochondrial genes in each sample. (D) Correlation analysis of total transcript number with mitochondrial genes, total gene number and PCA clustering analysis of all cells. [file Image_1.jpeg]

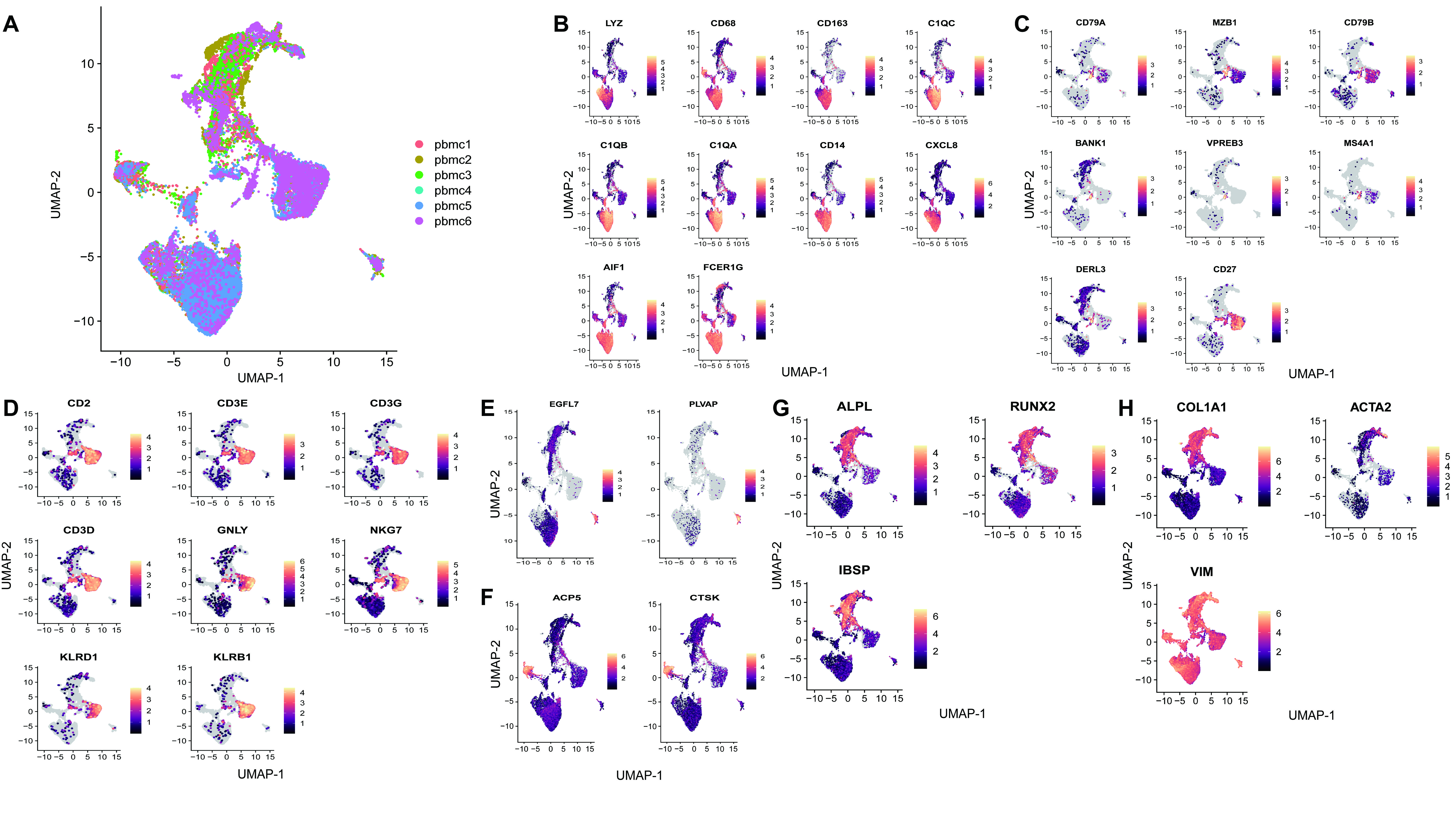

Supplement: Supplementary Figure 2 — (A) Two-dimensional visualization of cell distribution across samples. (B-H) The “umap” visualization of cell subpopulation-specific marker expression levels. [file Image_2.jpeg]

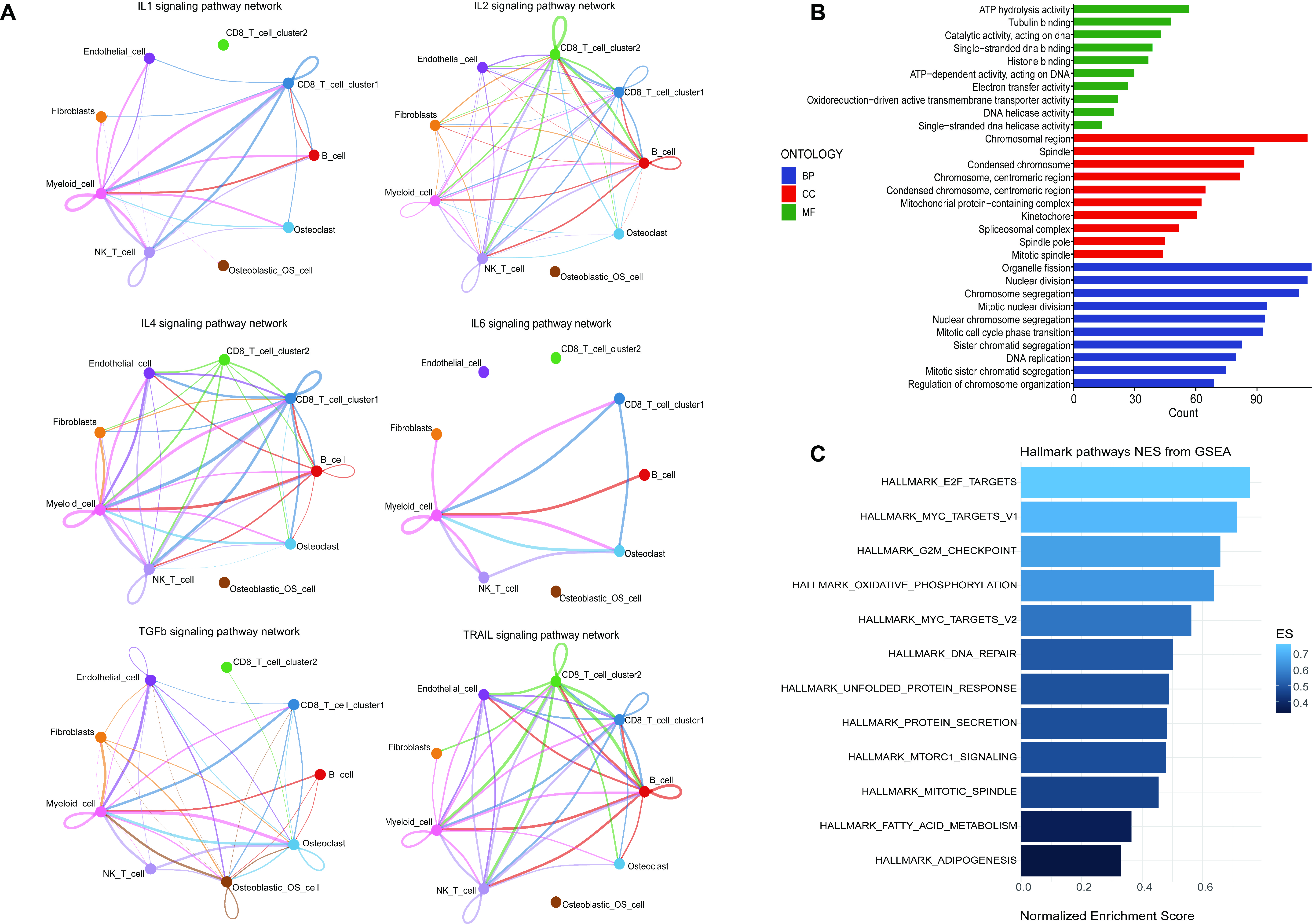

Supplement: Supplementary Figure 3 — (A) Communication networks among cell populations of multiple signaling pathways in cluster 2. (B) The enrichment of GO and GSEA terms in cluster 2. [file Image_3.jpeg]

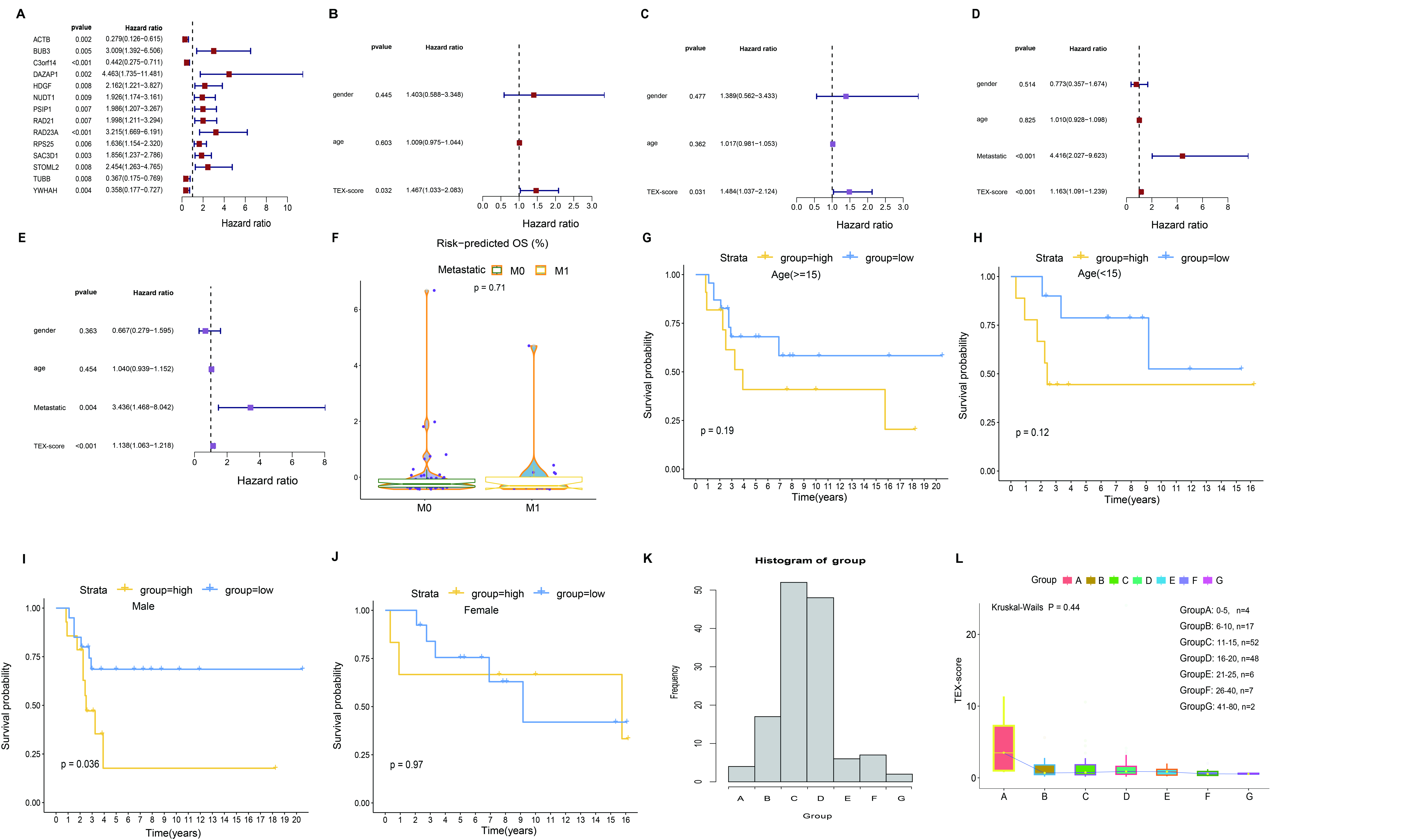

Supplement: Supplementary Figure 4 — (A) Risk coefficients for potential TEX-related genes calculated by univariate analysis. (B-E) The risk correlation analysis for each clinical characteristic and TEX-score in (B-C) The TARGETs and (D-E) the GSE21257 dataset. (F) Comparison of TEX scores of metastatic patients in the TARGETs cohort. (G-J) Kaplan–Meier survival analysis for OS patients with diverse clinical characteristics of age (G, H), and gender (I, J) in the GSE21257 dataset. (K-L) Distribution of TEX scores for all patients by age. [file Image_4.jpg]

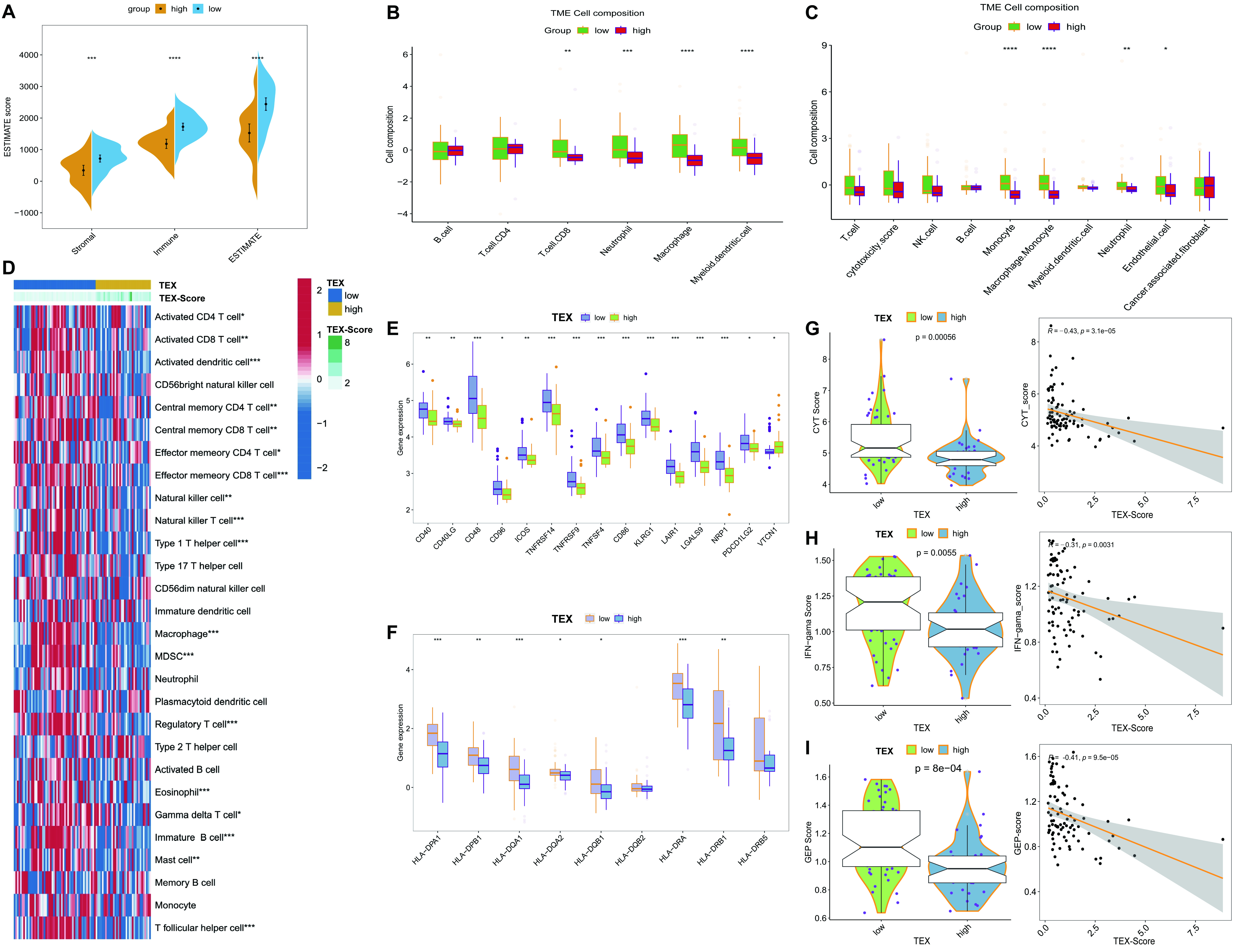

Supplement: Supplementary Figure 5 — (A-C) Immune characteristics related to the TEX-signature. (D) Heatmap showing the correlation between TEX-score and immune infiltrating. (E-F) The expression status of immune checkpoint and antigen-presenting molecules between low and high TEX-score groups. (G-I) The levels of CYT, IFN-γ and GEP between low and high TEX-score groups. [file Image_5.jpeg]

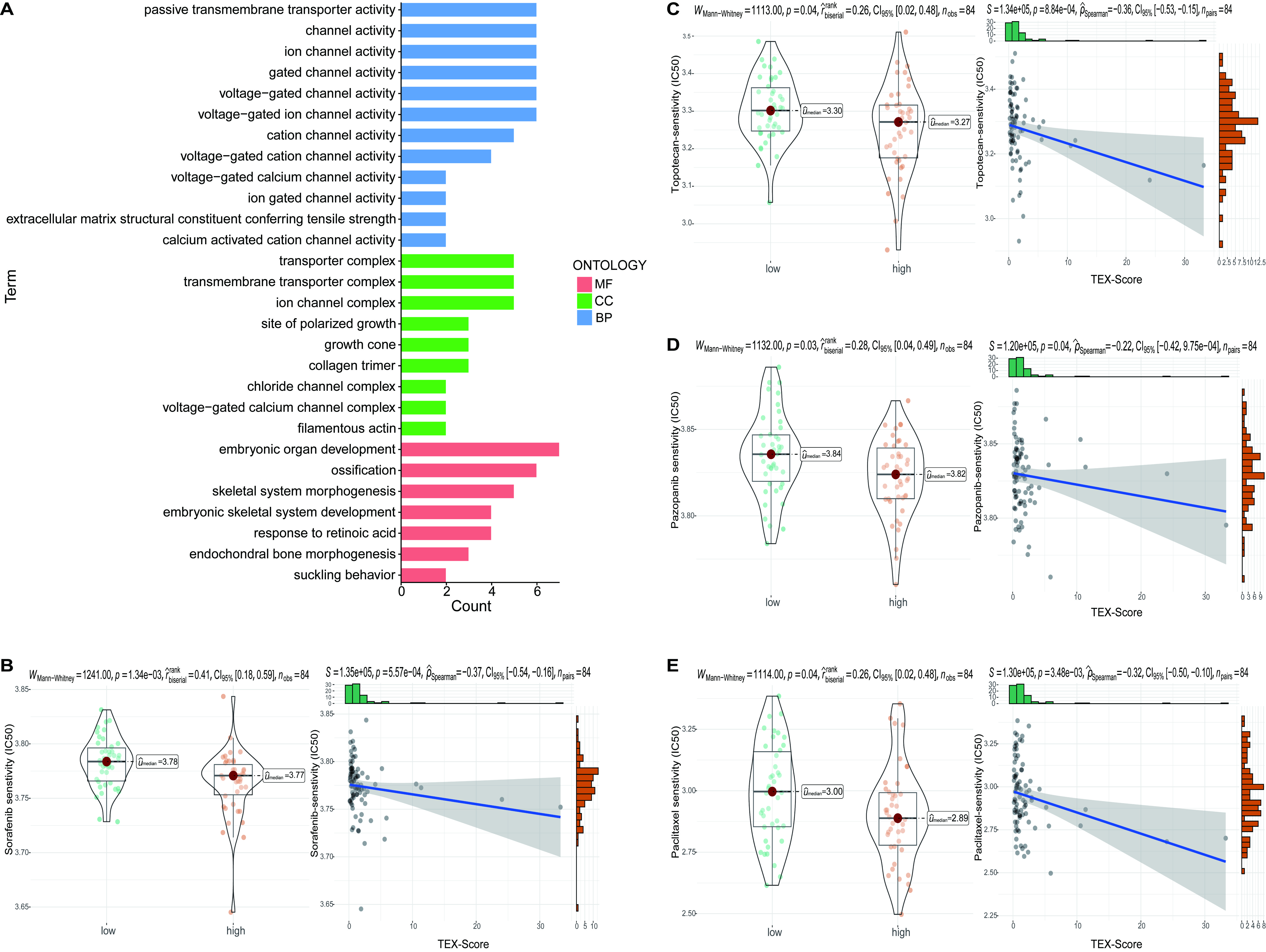

Supplement: Supplementary Figure 6 — (A) Function enrichment and metabolism of the high TEX-score. (B-E) The sensitivity of Sorafenib, Topotecan, Pazopanib, and Paclitaxel between low and high TEX-score. [file Image_6.jpeg]
